# Incidence and oncological implication of adrenal incidentalomas in esophageal cancer patients

**DOI:** 10.1093/dote/doad003

**Published:** 2023-01-31

**Authors:** J R van Doesburg, D M Voeten, M C Kalff, M I van Berge Henegouwen, S Jol, J E van den Bergh, A F Engelsman, S S Gisbertz, F Daams

**Affiliations:** Department of Surgery, Amsterdam UMC location Vrije Universiteit, Amsterdam, The Netherlands; Department of Surgery, Amsterdam UMC location University of Amsterdam, Amsterdam, The Netherlands; Department of Surgery, Amsterdam UMC location Vrije Universiteit, Amsterdam, The Netherlands; Department of Surgery, Amsterdam UMC location University of Amsterdam, Amsterdam, The Netherlands; Department of Surgery, Amsterdam UMC location University of Amsterdam, Amsterdam, The Netherlands; Cancer Treatment and Quality of Life, Cancer Center Amsterdam, Amsterdam, The Netherlands; Department of Surgery, Amsterdam UMC location University of Amsterdam, Amsterdam, The Netherlands; Cancer Treatment and Quality of Life, Cancer Center Amsterdam, Amsterdam, The Netherlands; Department of Radiology, Amsterdam UMC Location Vrije Universiteit, Amsterdam, The Netherlands; Cancer Treatment and Quality of Life, Cancer Center Amsterdam, Amsterdam, The Netherlands; Department of Radiology, Amsterdam UMC Location Vrije Universiteit, Amsterdam, The Netherlands; Department of Surgery, Amsterdam UMC location Vrije Universiteit, Amsterdam, The Netherlands; Cancer Treatment and Quality of Life, Cancer Center Amsterdam, Amsterdam, The Netherlands; Department of Surgery, Amsterdam UMC location University of Amsterdam, Amsterdam, The Netherlands; Cancer Treatment and Quality of Life, Cancer Center Amsterdam, Amsterdam, The Netherlands; Department of Surgery, Amsterdam UMC location Vrije Universiteit, Amsterdam, The Netherlands; Cancer Treatment and Quality of Life, Cancer Center Amsterdam, Amsterdam, The Netherlands

**Keywords:** adrenal incidentaloma, computer tomography (CT), esophageal cancer, positron emission tomography (PET)

## Abstract

Adrenal incidentalomas are regularly encountered during imaging for esophageal cancer patients, but their oncological significance remains unknown. This study aimed to describe the incidence and etiology of adrenal incidentalomas observed throughout the diagnostic workup. This retrospective cohort study included all esophageal cancer patients referred to or diagnosed in the Amsterdam UMC between January 2012 and December 2016. Radiology and multidisciplinary team meeting reports were reviewed for adrenal incidentalomas. In case of adrenal incidentaloma, the ^18^FDG-PET/CT was reassessed by a radiologist blinded for the original report. In case of a metachronous incidentaloma during follow-up, visibility on previous imaging was reassessed. Primary outcome was the incidence, etiology and oncological consequence of synchronous adrenal incidentalomas. This study included 1,164 esophageal cancer patients, with a median age of 66 years. Patients were predominantly male (76.1%) and the majority had an adenocarcinoma (69.0%). Adrenal incidentalomas were documented in 138 patients (11.9%) during the diagnostic workup. At primary esophageal cancer workup, 22 incidentalomas proved malignant. However, follow-up showed that four incidentalomas were inaccurately diagnosed as benign and three malignant incidentalomas were visible on staging imaging but initially missed. Stage migration occurred in 15 of 22 (68.2%), but this would have been higher if none were missed or inaccurately diagnosed. The oncological impact of adrenal incidentalomas in patients with esophageal cancer is significant as a considerable part of incidentalomas changed treatment intent from curative to palliative. As stage migration is likely, pathological examination of a synchronous adrenal incidentaloma should be weighted in mind.

## Introduction

Esophageal cancer rates continue to rise in the Western World.^1^^,^^2^ Esophagectomy with neoadjuvant chemo- and/or radiotherapy is the mainstay for curative treatment of esophageal cancer. However, determining the patients’ eligibility for surgery during the diagnostic workup is often complicated by the finding of an incidentaloma in the adrenal gland. In the general population, adrenal incidentalomas have a prevalence ranging from 0.2 to 7.0%.^3^ The differential diagnosis of adrenal incidentalomas includes a wide range of benign diseases, with the majority being lipid-ridge, non-hyperfunctioning adrenocortical adenomas.^4^ However, primary adrenocortical cancer as well as metastatic tumor deposits are also observed.^5^ Although the most classic pattern of metastasis for esophageal cancer comprises the liver, lungs, bones and brain,^6^^,^^7^ autopsy studies have also shown 3–12% of adrenal metastases to be of esophageal origin.^8^^,^^9^ Apart from their incidence, the oncological significance of adrenal incidentalomas detected during the diagnostic workup of esophageal cancer patients is currently not clear. This regularly leads to potentially unnecessary additional diagnostics which may impact hospital costs and increases time until treatment initiation. In addition, as the incidence of benign etiology among these adrenal incidentalomas might be considerable, these patients are potentially being exposed to unnecessary invasive examinations. Therefore, this study aimed to determine the incidence and etiology of adrenal incidentalomas, and the subsequent oncological impact of malignant adrenal incidentalomas.

## Methods

### Study Design

The first outcome of this study was the etiology and overall incidence of adrenal incidentalomas found synchronously to the diagnostic workup. Additional to this outcome, the proportion of synchronous malignant adrenal incidentalomas that resulted in stage migration for esophageal cancer treatment was determined. Endocrine consequences of adrenal etiology were not incorporated in this study. An adrenal incidentaloma was defined as an adrenal abnormality not previously known and detected during imaging performed for the diagnostic workup or follow-up of esophageal cancer.^10^ The current study was approved by the local ethical review board (2020.326) and patients were provided the opportunity to opt-out.

### Setting and Study Population

The study population comprised all patients with esophageal cancer and gastro-esophageal junction cancer, referred to or diagnosed in the Amsterdam UMC between 1 January 2012 and 31 December 2016. Patients were excluded when they met one of the following criteria: (1) referral to the Amsterdam UMC solely for a second opinion, (2) high-grade dysplasia or carcinoma in situ treated with endoscopic mucosal resection or submucosal dissection, (3) gastro-intestinal stroma tumor, adenosquamous carcinoma or neuroendocrine tumor, (4) presence of previously known adrenal abnormalities, (5) presence of abnormalities growing into the adrenal gland, originating from another structure, (6) patients with a reported adrenal incidentaloma which was not objectified during reassessment or (7) patients who applied for opt-out.

### Staging, Treatment and Follow-Up

Esophageal cancer staging was generally done using endoscopy with biopsy, ultrasound of the neck, cervico-thoraco-abdominal fluorodeoxyglucose-positron emission tomography (^18^FDG-PET)/ computed tomography (CT) or CT and endoscopic ultrasound. Staging was carried out in accordance with the 7th TNM classification of malignant tumors.^11^ All patients were discussed in a multidisciplinary team meeting including radiologists, pathologists, surgeons, medical oncologists, dieticians, nurses, physiotherapists and others. In general, patients eligible and fit for curative treatment received neo-adjuvant chemoradiotherapy followed by an esophageal resection. The first post-operative year, outpatient clinic visits were scheduled every 3 months. In the second to fourth post-operative year, follow-up was scheduled every 6 months. Thereafter, patients visited the outpatient clinic once more in the fifth postoperative year.^12^ Patients with potentially curable esophageal cancer ineligible for surgery received definitive chemoradiotherapy. When a recurrence was suspected, patients underwent an ^18^FDG-PET/CT, cervico-thoraco-abdominal CT scan or endoscopy on indication. Patients ineligible for curative treatment received palliative treatment or best supportive care. For those patients, the follow-up procedure was not standardized.

### Data Source and Variables

A retrospective cohort study of two tertiary referral centers. All radiological imaging and multidisciplinary team meeting reports of patients diagnosed with esophageal cancer were reviewed for adrenal incidentalomas from diagnosis of esophageal cancer until death or end of follow-up. Data on patient and tumor characteristics, disease specifications, radiological findings, whether the adrenal incidentaloma was mentioned during a multidisciplinary team meeting and whether additional diagnostics for further examination of the adrenal incidentaloma was performed, were extracted from the clinical patient files. Follow-up reports were evaluated for incidentalomas misdiagnosed as benign during the diagnostic workup. Adrenal incidentalomas were considered benign when (1) the adrenal gland showed no malignant characteristics on ^18^FDG-PET/CT (2) additional pathological assessment showed benign or (3) repeated imaging after at least 3 months showed no growth of the adrenal incidentaloma. Malignant characteristics included FDG-positivity on ^18^FDG-PET/CT, growth over time and greater than 10 Hounsfield Units on unenhanced CT combined with delay in contrast medium washout compared with adenomas.^13^

### Data Reassessment

When a synchronous adrenal abnormality was reported, the concerning ^18^FDG-PET/CT or CT scan was reassessed by a radiologist (blinded for the original report). The reassessment comprised the size and location within the adrenal gland, and in case an ^18^FDG-PET/CT was available, the presence of FDG-avidity. As ^18^FDG-PET/CT was not available for all patients, the ^18^FDG-PET/CT scans were re-evaluated for the visibility of the adrenal incidentaloma without ^18^FDG. When metachronous adrenal incidentalomas were encountered during follow-up, preceding imaging was reassessed to see if the incidentaloma was, in retrospect, already visible on initial staging imaging.

### Statistics

Descriptive statistics were based on all subjects in the analytical cohort, and patients with adrenal incidentalomas. Continuous data were tested for normality using histograms. In case of a skewed distribution, medians with interquartile ranges (IQR) were reported. Categorical data were shown in tables as absolute numbers and in fractions (%). Missing values were not counted when calculating proportions. Data were stored and analyzed using IBM SPSS Statistics, Version 26.0. Armonk, 2019.

## Results

### Population

**Fig. 1:**
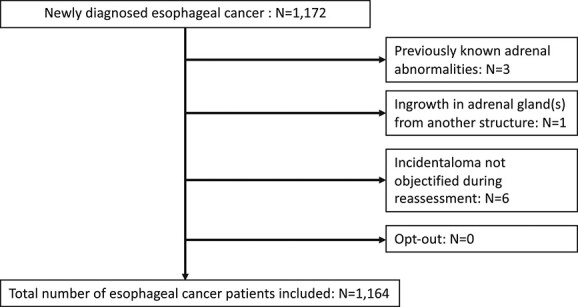
Flow chart of patient selection.

In total, 1,164 esophageal cancer patients were included (Fig. 1). Most were diagnosed with a cT3 tumor (64.3%), and one or two suspected regional lymph node metastases (cN1; 36.0%). The majority of patients (75.8%) had no distant metastasis (cM0) upon diagnosis. Table 1 shows the baseline characteristics of the included patients.

**Table 1:** Baseline characteristics of all patients, patients with an adrenal incidentaloma and patients with a malignant adrenal incidentaloma.

		**All patients N = 1,164**		**Adrenal incidentaloma N = 141**		**Benign adrenal incidentaloma N = 110**		**Malignant adrenal incidentaloma N = 26**		**Incidentaloma with unclear etiology *N* = 2**	
		***N***	**%**	***N***	**%**	***N***	**%**	***N***	**%**	***N***	**%**
**Sex**	Male	886	76.1	103	73.0	79	71.8	20	76.9	2	100
**Age at diagnosis**	Median (IQR)	66.0 (60.0–73.0)		67.0 (62.0–72.5)		68.0 (62.0–73.0)		67.0 (61.8–71.0)		69.0 (62.0–69.0)	
**Comorbidities**	Pulmonary	216	18.6	30	21.4	23	20.9	7	26.9	-	-
	Cardiac	298	25.7	37	26.4	30	27.3	5	19.2	1	50.0
	Vascular	474	40.8	68	48.6	53	48.2	12	46.2	2	100
	Diabetes	167	14.4	29	20.7	23	20.9	3	11.5	2	100
**ASA-class**	ASA 1	152	27.5	13	20.6	11	20.0	2	7.7	-	-
	ASA 2	291	52.7	33	52.4	28	50.9	3	11.5	-	-
	ASA 3	107	19.4	16	25.4	15	27.3	1	3.8	-	-
	ASA 4	2	0.4	1	1.6	1	1.8	-	-	-	-
**Workup**	^18^FDG-PET/CT	763	66.5	96	68.1	79	71.8	14	53.8	-	-
	CT	1142	99.5	140	99.3	109	99.1	26	100	2	100
	US	784	68.3	93	66.0	73	66.4	17	65.4	1	50.0
	EUS	802	69.9	92	65.2	75	68.2	15	57.7	-	-
	EBUS	64	5.6	6	4.3	4	3.6	-	-	1	50.0
**Year of diagnosis**	2012	190	16.3	22	15.6	19	17.3	3	11.5	-	-
	2013	210	18.0	25	17.7	17	15.5	5	19.2	1	50.0
	2014	240	20.6	34	24.1	24	21.8	9	34.6	-	-
	2015	271	23.3	29	20.6	25	22.7	4	15.4	-	-
	2016	253	21.7	31	22.0	25	22.7	5	19.2	1	50.0
**Histology**	Adenocarcinoma	801	69.0	104	73.8	79	71.8	21	80.8	1	50.0
	Squamous cell carcinoma	350	30.1	35	24.8	30	27.3	5	19.2	-	-
	Small cell carcinoma	9	0.8	1	0.7	1	0.9	-	-	-	-
	Large cell carcinoma	1	0.1	1	0.7	-	-	-	-	1	50.0
**Primary tumor location**	Proximal	68	5.9	7	5.0	6	5.5	-	-		
	Mid	170	14.7	13	9.2	10	9.1	3	11.5		
	Distal	746	64.3	86	61.0	65	59.1	18	69.2	1	50.0
	GEJ/Cardia	167	14.4	33	23.4	27	24.5	5	19.2	1	50.0
	Multifocal	9	0.8	2	1.4	2	1.8	-	-		
**cT**	T1	49	4.5	6	4.7	5	4.8	1	4.8	-	-
	T2	177	16.4	23	17.8	18	17.3	4	19.0	-	-
	T3	683	63.4	83	64.3	68	65.4	13	61.9	1	100
	T4	72	6.7	9	7.0	6	5.8	2	9.5	-	-
	Tx	96	8.9	8	6.2	7	6.7	1	4.8	-	-
**cN**	N0	311	28.7	42	31.1	38	34.9	3	13.6	1	50.0
	N1	389	36.0	43	31.9	38	34.9	3	13.6	-	-
	N2	266	24.6	37	27.4	25	22.9	11	50.0	1	50.0
	N3	71	6.6	9	6.7	6	5.5	3	13.6	-	-
	N+	9	0.8	1	0.7	-	-	1	4.5	-	-
	Nx	36	3.3	3	2.2	2	1.8	1	4.5	-	-
**cM**	M0	870	75.8	117	83.0	99	90.0	14	53.8	2	100
	M1	179	15.6	18	12.8	8	7.3	10	38.5	-	-
	Mx	99	8.6	6	4.3	3	2.7	2	7.7	-	-

IQR, interquartile range; ASA-class, American Society of Anesthesiologists Physical Status Classification System ^18^;FDG-PET, fluorodeoxyglucose-positron emission tomography; CT, computed tomography; EUS, endoscopic ultrasound; EBUS, endobronchial ultrasonography; GEJ, gastro-esophageal junction; cT, clinical primary tumor stage; cN, clinical regional lymph nodes stage; cM, clinical distant metastasis stage, TNM classification of malignant tumors, 7th edition.

### Patients with Adrenal Incidentaloma

**Fig. 2:**
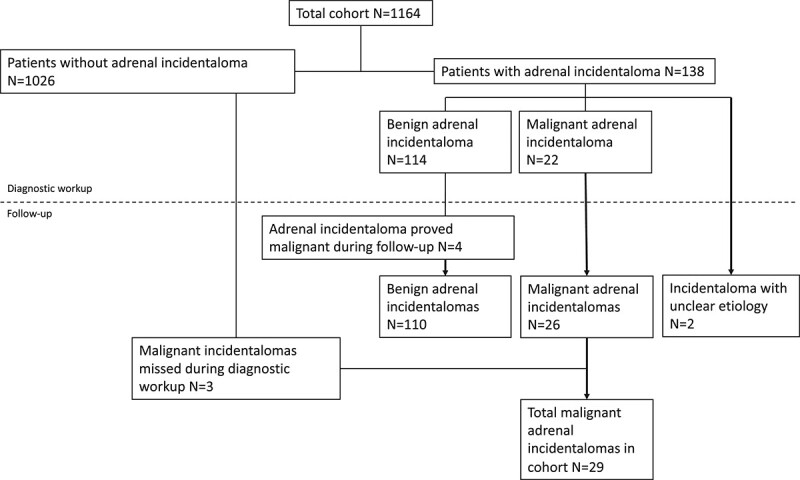
Flow chart of adrenal incidentalomas and corresponding etiology, during diagnostic workup and during follow-up.

During the diagnostic workup, an adrenal incidentaloma was observed in 138 of 1,164 included patients (11.9%). In 114 (82.6%) of the patients with an adrenal incidentaloma, the incidentaloma was considered benign (Fig. 2). Four of those, however, proved malignant upon follow-up. Table 2 shows the etiology of all incidentalomas according to the primary assessment during diagnostic workup, most frequently being adenomas (60.9%). In six of the patients (4.3%) with benign adrenal incidentalomas, the exact etiology of the adrenal incidentaloma was undefined. In 22 of the 138 patients with adrenal incidentalomas (15.9%), the origin was considered malignant (overall incidence: 22 out of 1,164; 1.9%). In 19 of the 22 patients with a probable malignant adrenal incidentaloma, the etiology was considered esophageal metastasis but in the remaining three a primary pheochromocytoma could not be ruled out. In two patients, the incidentaloma did not meet the criteria for benignity nor malignity and no biopsy was conducted to clarify etiology.

**Table 2:** Adrenal incidentaloma etiology according to primary assessment at diagnostic workup

		**Adrenal incidentalomas N = 138**	
		**N**	**%**
**Benign etiology**	Benign	114	82.6
	Adenoma(s)	84	60.9
	Cyst(s)	3	2.2
	Hyperplasia	21	15.2
	Undefined nodular nonconformity	6	4.3
**Malignant etiology**	Malignant	22	15.9
	Metastases	19	13.8
	Metastases or pheochromocytoma	3	2.2
**Unclear etiology**		2	1.5

### Diagnostic Findings

An ^18^FDG-PET/CT was conducted for 108 of the 138 patients with an adrenal mass, in all patients the adrenal incidentaloma was visible on both ^18^FDG-PET/CT and CT alone. Twenty-one (22.6%) showed FDG avidity. From the 22 patients with a malignant mass, 14 underwent ^18^FDG-PET/CT. Of those, nine (64.3%) showed FDG avidity. In 35 of 138 patients (25.4%) with an adrenal incidentaloma, additional diagnostics to characterize the adrenal incidentaloma were performed (Table 3). An adrenal CT was performed in 28 patients, in seven patients this was followed by a biopsy, of which five suggested malignancy. Of those 28 patients, two patients initially only received an adrenal CT, but underwent an additional adrenal CT with biopsy as the results of the primary adrenal CT were inconclusive. In total, for seven out of 35 patients that underwent additional diagnostics, the adrenal incidentaloma proved malignant. Overall, the finding of a malignant adrenal incidentaloma during the diagnostic workup resulted in stage migration for 15 out of 22 patients (68.2%), and changed policy from curative intent to palliative care in eight (36.4%). For the other eight patients, the policy was already palliative due to clinical stage or due to other metastatic sites discovered at the same time.

**Table 3:** Adrenal incidentaloma characteristics

		**Adrenal incidentalomas N = 141**		**Benign adrenal incidentaloma N = 110**		**Malignant adrenal incidentalomas N = 26**		**Incidentaloma with unclear etiology N = 2**	
		***N***	**%**	***N***	**%**	***N***	**%**	***N***	**%**
FDG avidity^*^	Yes	21	22.6	12	15.2	9	64.3	-	-
	No	75	78.1	67	84.8	5	35.7	-	-
**Laterality**	Left	76	53.9	59	53.6	12	46.2	2	100
	Right	27	19.1	24	21.8	3	11.5	-	-
	Both	38	27.0	27	24.5	11	42.3	-	-
**Position**	Medial	20	15.2	15	14.9	5	19.2	-	-
	Lateral	20	15.2	17	16.8	3	11.5	-	-
	Body	74	56.1	56	55.4	14	53.8	2	100
	Diffuse/Multifocal	18	13.6	13	11.8	4	15.3	-	-
**Size (mm)**	Median (IQR)	18.0 (13.0–22.5)		17.0 (13.0–21.0)		21.0 (16.0–30.5)		27.0 (26.0–27.0)	
Second AI^*^^*^	Median (IQR)	13.0 (10.0–19.0)		13.0 (10.0–19.5)		12.5 (10.5–19.0)		-	
**AI mentioned at MDT meeting**	Yes	77	54.6	54	49.1	21	80.8	2	100
	No	61	45.4	56	50.9	5	19.2	-	-
**Additional diagnostics**	Yes	35	24.8	24	21.8	11	42.3	-	-
	No	106	75.2	86	78.2	15	57.7	2	100
**Type of additional diagnostics** ^*^ ^*^ ^*^	CT adrenal	21	60.0	19	79.2	2	18.2	-	-
	CT + biopsy	9	25.7	2	8.3	7	63.6	-	-
	MRI	4	11.4	3	12.5	1	9.0	-	-
	PET-CT	2	5.7	-	-	2	18.2	-	-
	Endocrinological	1	2.9	1	4.2	-	-	-	-
**Stage upon AI discovery** ^*^ ^*^ ^*^ ^*^	Stage 1	11	7.9	10	9.3	1	3.8	-	-
	Stage 2	32	23.0	27	25.0	3	11.5	1	50.0
	Stage 3	77	55.4	60	55.6	14	53.8	1	50.0
	Stage 4	19	13.7	11	10.2	8	30.8	-	-

AI, adrenal incidentaloma, ^18^FDG-PET, fluorodeoxyglucose-positron emission tomography; CT, computed tomography; FDG+, fluorodeoxyglucose avidity; IQR, interquartile range; MDT, multidisciplinary team.

^*^The percentage of FDG avidity was calculated based on the patients that underwent ^18^FDG-PET/CT during the diagnostic workup (see Table 1), not all patients in the cohort underwent ^18^FDG-PET/CT.

^^*^^*^^In case a patient had multiple incidentalomas, the largest and the second largest was analyzed.

^^*^^*^^*^^Some patients underwent multiple types of additional diagnostics.

^^*^^*^^*^^*^^For the three incidentalomas missed during the diagnostic workup, the disease stage during the workup, when the adrenal gland was in retrospect already visible, was counted.

### Reassessment of Synchronous Adrenal Incidentalomas

Of all 114 patients with an adrenal incidentaloma classified as benign during the diagnostic workup, four (3.5%) showed to be malignant during follow up. Meaning only 110 of the incidentalomas found during the diagnostic workup were actually benign (79.7%). For two patients, the discovery of the malignant origin of the adrenal incidentaloma resulted in stage migration from Stage 3 to Stage 4. For the other two patients, the malignant etiology was revealed only when patients already had disseminated disease at different sites. If these adrenal incidentalomas would have been identified as malignant during the initial diagnostic workup, treatment intent would have altered from curative intent to palliative intent in all four patients.

### Reassessment of Metachronous Adrenal Incidentalomas

In addition to the 138 adrenal incidentalomas discovered synchronous to the diagnostic workup, 43 metachronous incidentalomas were discovered. Of those, 14 (32.6%) proved malignant. Of these malignant incidentalomas, three were, in retrospect, already visible on prior scans, but missed during the initial diagnostic workup and were all allocated to curative treatment. If these adrenal incidentalomas would have been identified as malignant during the initial diagnostic workup, treatment intent would have altered from curative intent to palliative intent.

## Discussion

This study investigated the incidence of adrenal incidentalomas encountered during imaging performed for esophageal cancer. Adrenal incidentalomas were observed in 138 out of 1,164 patients (11.9%). Of those, 22 were diagnosed as malignant during the initial workup (15.9%). In 15 of 22 patients (10.9%) this resulted in stage migration and in 8 (5.8%) a change in treatment intent from curative intent to palliative care. In addition, four incidentalomas were misdiagnosed as benign during the diagnostic workup but proved malignant during follow-up and three malignant adrenal incidentalomas were missed during the diagnostic workup. Including the incidentalomas missed or inaccurately diagnosed, adrenal incidentalomas were synchronously present in 141 out of 1,164 (12.1%) patients and malignant adrenal incidentalomas in 29 out of 1,164 (2.5%) included patients with esophageal cancer.

As adrenal incidentalomas found during the diagnostic workup were frequently of malignant origin, establishing adequate management for adrenal incidentaloma patients is highly important. In addition, as adrenal incidentalomas often change treatment intent from curative intent to palliative care, taking measures to prevent misdiagnoses or missed incidentalomas is important, to avoid patients receiving unsuitable care.

No literature regarding adrenal incidentalomas during diagnostic imaging of esophageal cancer patients is available. Studies on the incidence of adrenal incidentalomas in patient without cancer vary from 2.3 to 5.9%.^3^^,^^14–17^ This is lower than the 11.9% found in the current study. However, the numbers found in these studies could be an underestimation of the incidence in this studies particular research group, as metastases are a lot less common when patients with extra-adrenal cancer are excluded. Moreover, multiple studies have correlated the prevalence of adrenal nodules to increase with age, from 0.2% in young patients to up to over 14% in subjects older than 70 years.^15^^,^^18–21^ As the population in this study has a median age of 66, more incidentalomas are to be expected than in studies conducted in the general population. Very few studies are available on the incidence of malignant incidentalomas amongst patients with known extra-adrenal cancer. Studies in patients with non-small cell lung carcinoma have shown that adrenal metastases were present in 5–10% of patients at initial presentation.^22^^,^^23^ This is higher than the 2.5% of adrenal metastases we found in our study population. It is known, however, that esophageal cancer is a lot less likely to spread to the adrenal gland then lung cancer.^9^

Malignant characteristics may not always be distinctively present on the initial ^18^FDG-PET/CT. Therefore, follow-up should be considered even when the adrenal incidentaloma appear benign at presentation. An imaging algorithm, as developed by McNicholas *et al*., could be used to determine whether or not to conduct additional imaging or biopsy.^24^ This imaging algorithm uses CT and chemical-shift MR imaging for the characterization of adrenal masses in patients with primary malignancy and no other evidence of metastatic disease. Based on Hounsfield units and the adrenal signal intensity normalized to that of the spleen, recommendations for biopsy or follow-up imaging are made.

To our knowledge, this is the first study reporting on the incidence of adrenal incidentalomas and investigating their oncological significance in esophageal cancer patients. As patients with obvious metastatic disease are often not referred to the tertiary center, the incidence of adrenal metastases may be underestimated. A limitation of this study is that as it is a retrospective study, biopsies were not available for all patients. Therefore, only seven out of 19 adrenal incidentalomas considered a metastasis were confirmed with biopsy. ^18^FDG-PET scans were also not available for all patients, which could affect the incidence and interpretation of results. However, in this study, all incidentalomas found on ^18^FDG-PET/CT were also visible on CT scan alone. Hence, the effect of under-utilization of ^18^FDG-PET is likely to be limited. Therefore, it was not always possible to distinguish metastases from primary adrenal carcinomas. Another limitation of this study is that it was focused solely on the oncological implication of adrenal incidentalomas, the impact of pheochromocytomas and other benign adrenal diseases was not assessed and it was not possible to assess the role of endocrinological assessment in diagnosing adrenal incidentalomas. Therefore, future research should focus on radiological imaging analysis, the use of endocrinological assessment and on establishing adequate management for adrenal incidentaloma patients.

This study demonstrated that esophageal metastasis to the adrenal gland can occur in patients without disseminated disease and regularly causes a change in policy from curative intent to palliative. Image analysis by a radiologist experienced in assessing adrenal incidentalomas is required, as this study showed that clinically relevant adrenal incidentalomas might be missed or misdiagnosed. It is therefore recommended to conduct additional diagnostics after radiological evaluation to establish the proper etiology of the adrenal incidentaloma. Adrenal incidentalomas should be monitored for growth and radiological changes, even if they seem benign and nonfunctional at initial evaluation.

## Data Availability

The data that support the findings of this study are not publicly available due to their containing information that could compromise the privacy of research participants, but are available from one of the corresponding authors [J.R. van Doesburg or F.Daams] upon reasonable request.
